# Prescriber and staff perceptions of an electronic prescribing system in primary care: a qualitative assessment

**DOI:** 10.1186/1472-6947-10-72

**Published:** 2010-11-19

**Authors:** Emily Beth Devine, Emily C Williams, Diane P Martin, Dean F Sittig, Peter Tarczy-Hornoch, Thomas H Payne, Sean D Sullivan

**Affiliations:** 1 Pharmaceutical Outcomes Research and Policy Program, University of Washington, Box 357630, Seattle, WA 98195-7630, USA; 2 Department of Medical Education and Biomedical Informatics, University of Washington, Box 357240, Seattle, WA 98195-7240, USA; 3 Health Services Research & Development, Veterans Affairs Puget Sound Health Care System, 1100 Olive Way, Suite 1400, Seattle, WA 98101, USA; 4 Department of Health Services, University of Washington, Box 357660, Seattle, WA 98195-7660, USA; 5 School of Health Information Sciences, University of Texas, Houston, UT-Memorial Hermann Center for Healthcare Quality & Safety, 6410 Fannin Street, Houston, TX 77030, USA; 6 Department of Medicine, University of Washington, Box 359968, Seattle, WA 98195-9968, USA

## Abstract

**Background:**

The United States (US) Health Information Technology for Economic and Clinical Health Act of 2009 has spurred adoption of electronic health records. The corresponding meaningful use criteria proposed by the Centers for Medicare and Medicaid Services mandates use of computerized provider order entry (CPOE) systems. Yet, adoption in the US and other Western countries is low and descriptions of successful implementations are primarily from the inpatient setting; less frequently the ambulatory setting. We describe prescriber and staff perceptions of implementation of a CPOE system for medications (electronic- or e-prescribing system) in the ambulatory setting.

**Methods:**

Using a cross-sectional study design, we conducted eight focus groups at three primary care sites in an independent medical group. Each site represented a unique stage of e-prescribing implementation - pre/transition/post. We used a theoretically based, semi-structured questionnaire to elicit physician (n = 17) and staff (n = 53) perceptions of implementation of the e-prescribing system. We conducted a thematic analysis of focus group discussions using formal qualitative analytic techniques (i.e. deductive framework and grounded theory). Two coders independently coded to theoretical saturation and resolved discrepancies through discussions.

**Results:**

Ten themes emerged that describe perceptions of e-prescribing implementation: 1) improved availability of clinical information resulted in prescribing efficiencies and more coordinated care; 2) improved documentation resulted in safer care; 3) efficiencies were gained by using fewer paper charts; 4) organizational support facilitated adoption; 5) transition required time; resulted in workload shift to staff; 6) hardware configurations and network stability were important in facilitating workflow; 7) e-prescribing was time-neutral or time-saving; 8) changes in patient interactions enhanced patient care but required education; 9) pharmacy communications were enhanced but required education; 10) positive attitudes facilitated adoption.

**Conclusions:**

Prescribers and staff worked through the transition to successfully adopt e-prescribing, and noted the benefits. Overall impressions were favorable. No one wished to return to paper-based prescribing.

## Background

For years, the Institute of Medicine has championed the use of electronic health records (EHRs) as a way to improve the quality, safety, and efficiency of the United States (US) healthcare system. [1-7] Yet data from a national survey conducted in 2007-2008 revealed that only 4% of physicians practicing in the ambulatory setting reported using a fully functional EHR. [8] The Health Information Technology for Economic and Clinical Health Act has since provided needed impetus to spur adoption. The Office of the National Coordinator for Health information Technology (HIT) has set initial standards, specifications, and certification criteria for EHRs. [9] The Centers for Medicare and Medicaid Services has defined what constitutes 'meaningful use'. [10]

Topping the proposed meaningful use list, and necessary for certification, is the computerized provider order entry (CPOE) system to order medications, laboratory tests, procedures, images, and referrals. [10] Yet, a recent review of CPOE implementation in hospitals in seven Western countries (Australia, France, Germany, the Netherlands, Switzerland, United Kingdom, and US) reveals that implementation is slow, with adoption rates of 20% or less. [11] Integration to existing EHRs is problematic; indeed, in these countries, the use of EHRs is also rare. [11,12] These investigators found no relationship between health care system organization and CPOE implementation, noting that the cost burden of implementation often rests with the hospitals, despite the existence of national or regional incentives for adoption. Issues of professional autonomy, identity and conflict remain as barriers. In this environment, examples of successful CPOE adoption are critically important. To date, most examples are from academic medical centers, using homegrown systems; fewer reports are from the ambulatory setting. [13-18] The work of one team of researchers - the physician order entry team (POET) - describes a successful implementation in the ambulatory setting of a health maintenance organization (HMO). [19-21] To our knowledge, a detailed description of the perceptions of physicians that have successfully implemented a CPOE system in an independent medical group practice has not been published.

We implemented CPOE software for medication ordering - an electronic (e-) prescribing system - in an independent medical group. Clinic leadership motivated the implementation by highlighting the expected improvements in medication safety and efficiencies associated with use of fewer paper charts. We have published a collection of studies that describes the impact of this implementation on medication errors and adverse drug events [22], on the attitudes of prescribers and staff toward e-prescribing adoption [23], and on the time-intensity of e-prescribing [24]. We have also narrated the lessons we learned that enabled successful adoption [25]. In this companion manuscript we describe the results of a series of focus groups we conducted with physicians and staff across three primary care sites in varying stages of e-prescribing implementation. Our primary objective was to describe their perceptions, to note what worked well, what needed improvement, and what contributed to successful implementation. Our secondary objective was to map our findings to a theoretical model that describes information technology (IT) adoption - the Information Technology Adoption Model (ITAM). [26-28]

### Theoretical Model

**Figure 1:**
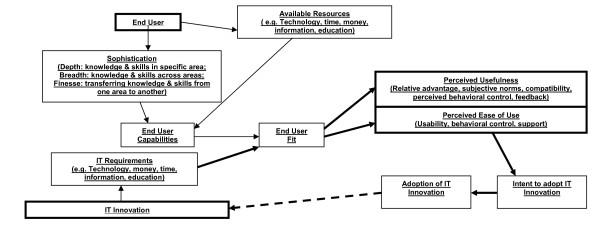
**Information Technology Adoption Model (ITAM)**.

The ITAM was developed by Dixon. [26-28] It is based on Davis' Technological Acceptance Model that specifies that *perceived usefulness *and, to a lesser extent, *perceived ease of use*, influence an individual's attitude toward adoption. [29,30] (Figure 1) The ITAM begins with two inputs - the *End User *and the *IT Innovation *(bold boxes). The end user brings his/her personal characteristics and level of sophistication with computer use (depth, breadth, and finesse of knowledge and skills). (S)he also brings *Available Resources*, such as education. Together these comprise *End User Capabilities*. The second input, the *IT Innovation*, consists of *IT Requirements*, similar to the user's *Available Resources*. Together, these two inputs determine *End User Fit. Fit *then determines *Perceived Usefulness *and *Perceived Ease of Use *(bold boxes), which, in turn, determine *Intent to Adopt *and *Adoption. Perceived Usefulness *represents the constructs of relative advantage (how useful the innovation is, compared to the alternative), subjective norms (the sociocultural environment), compatibility (consistency of the innovation to users' values and experiences), and feedback (the ability of users to determine the outcome of implementation). *Perceived Ease of Use *represents the constructs of usability (affecting change by adopting the innovation), perceived behavioral control (how easy/difficult it is to adopt), and support (support available during implementation).

Dixon used the ITAM to study IT adoption in the primary care setting. [28-30] We use it to provide the theoretical underpinning for elicitation of end user perceptions of an e-prescribing system. Our end users are the prescribers and staff. We enhance the model in proposing that, as leadership receives feedback from end users, system improvements are made, end users' perceptions modified, and adoption increased. Our enhancement is the link between adoption and IT Requirements, which we represent with a bold, dotted arrow. We represent the continuous improvement loop with bold arrows.

## Methods

### Setting

We conducted the study at The Everett Clinic, the largest independent medical group in Washington State. Physician-owners care for 275,000 patients in sixty clinics, at fourteen sites within a twenty-mile radius in the north Puget Sound area. The Everett Clinic logs 650,000 ambulatory visits and clinicians write 2.7 million prescriptions annually. HIT staff began development of a homegrown EHR in 1995. Early on, the EHR was comprised of electronic chart notes, laboratory values, and imaging reports. The e-prescribing module was rolled out from 2003-2005 and interfaces with the existing EHR. The e-prescribing system is web-based and uses point-and-click functionality. The system makes use of the drug database from Multum™ (Cerner, Denver, CO). It generates new and renewed prescriptions. Prescribers select medications from pull-down menus or from 'favorites' lists. Directions can be selected or typed as free-text. During the study the e-prescribing system included only basic dosing guidance; duplicate therapy checks; and, when the clinician enters a child's weight, weight-based, pediatric dosing of drug, strength, and bottle size (if liquid medication). Allergy, drug-drug interaction, drug-disease interaction, and laboratory monitoring alerts were added after completion of data collection. Clinic staff (nurses and medical assistants) enter prescriptions into a queue for review; only licensed prescribers can authorize and release these to a pharmacy of the patient's choice.

E-prescribing implementation was initiated first at primary care clinic sites where providers were eager to embrace the technology, the rationale being that early successes would set a positive example for those reluctant to adopt. Use was initially voluntary. Implementation in specialty clinics took place subsequent to study completion. Clinic-wide use was eventually mandated. Initially, physicians accessed the EHR via desktop PCs in their personal offices or at shared workstations; staff accessed solely from workstations. Shortly after e-prescribing rollout, The Everett Clinic made the strategic decision to enhance EHR access by installing a PC in each of 505 examination rooms. A portable laptop was provided to each physician as an interim step toward this end.

### Study Design

To capture information about physicians' and staff (participants) perceptions of the e-prescribing system, we conducted a total of eight focus groups at three primary care sites during 2005-2006. Each site consisted of a family practice, internal medicine, pediatric, and walk-in clinic. Our primary objective was to elicit information about and describe perceptions of primary care prescribers and staff across the continuum of stages of implementation (pre/transition/post) and degrees of eagerness to adopt (reluctant/eager). Due to their reluctance, Site C was the last to adopt, and implementation occurred during the course of this study. Those at site B were willing, and those at site A were eager. Implementation at sites A and B took place twelve months prior to this study. Those at site A used portable laptops during data collection, as data collection took place prior to installation of a PC in each examination room. (Table 1) We conducted two focus groups at Site A and two at Site B - one at each site with physicians and a separate one with staff. We conducted three focus groups at site C - one with physicians, and two with staff, one before and a second six-months after implementation, the latter representing the transition phase. To gain additional insight from staff that experienced implementations at multiple sites, our eighth focus group was comprised of staff from a 'float pool', whose work crossed sites. The cross-sectional design of the study is depicted in Table 2.

**Table 1:** Timing of focus groups, description of hardware and software, and characteristics of participants

	Site C		Site B	Site A	Float Pool
**Focus Group Date**	**March 2005 ** **(Pre-implementation)**	**October 2005** **(Transition)**	**June/September 2005** **(Post-** **Implementation)**	**June 2006** **(Post-Implementation)**	**April 2005** **(variable stages of implementation)**

***Description of Hardware and Software***					

**Hardware configuration at time of focus group**	Basic*	Basic*	Basic*	Basic* plus Prescribers using portable laptops since June 2005 as step toward having PC in exam room	Basic* plus Variable
**Software in use at time of focus group**	Basic** plus e-rx system absent; paper-based prescribing only	Basic** plus e-rx system in place since April 2005	Basic** plus e-rx system in place since July 2004	Basic** plus e-rx system in place since August 2004	Basic** plus e-rx system variable

***Characteristics of Participants***					

**Participants**	Focus Group 1: 8 Physicians	No physician focus group conducted	Focus Group 4: 6 Physicians	Focus Group 6: 3 Physicians	
	Focus Group 2: 8 Staff	FocusGroup3: 7 Staff	Focus Group 5: 12 Staff	Focus Group 7: 4 Staff	Focus Group 8: 22 Staff
**Number employed at site; proportion participating in focus group**	Physicians: 10; 80% (8/10)	Physicians: N/A	Physicians: 15; 40% (6/15)	Physicians: 8; 38% (3/8)	N/A
	Staff: 17; 48% (8/17)	Staff 17; 41% (7/17)	Staff: 25; 48% (12/25)	Staff: 11; 36% (4/11)	

*Basic hardware configuration = Desktop computers in prescriber offices and at clinic workstations for ten years; **Basic software = Homegrown electronic health record in place for ten years; e-rx = electronic prescribing; N/A = not applicable

**Table 2:** Cross-sectional study design

	Pre-Implementation	Transition	Post-Implementation
**Site C**	One focus group for prescribers; One focus group for staff	One focus group for staff	-

**Site B**	-	-	One focus group for prescribers; One focus group for staff

**Site A**	-	-	One focus group for prescribers; One focus group for staff

**Float Pool**	←One focus group for staff→		

Our secondary objective was to map our findings to the ITAM. [26-28] To facilitate this, we conducted a literature search to identify themes associated with CPOE or e-prescribing implementation identified by other investigators. [31-46] We extracted themes that explained both positive and negative aspects of CPOE use, and mapped these to the ITAM. The results of this *a priori *mapping exercise are available from the authors. We used this theoretical model, informed by the current literature, to construct our semi-structured elicitation tool, the Focus Group Questionnaire. [47] (Table 3) Despite the authors' foregone knowledge of the potential benefits and challenges associated with CPOE use, to prevent the introduction of bias by the authors we intentionally designed the questionnaire using very broad terms. The University of Washington Human Subjects Committee approved all research activities.

**Table 3:** Semi-Structured Focus Group Questionnaire [31-47]

**First Topic Area:**	
**Expectations of how the e-prescribing system will help you in your professional life**	
**Site C**	"How do you expect the e-prescribing system to help you in your practice?"
	
**Sites A and B**	"Have your expectations been met for how the e-prescribing system would help you in your practice?"
	"Are there positive features of the e-prescribing system that you have identified since implementation?"
**Second Topic Area:**	
**Concerns and Fears about the e-prescribing system**	
**Site C**	"What are your concerns and fears about the implementation and use of the e-prescribing system?"
**Sites A and B**	"Were your concerns and fears about the e-prescribing system realized?"
	"Are there new concerns or fears that have arisen that were not identified prior to implementation?"
**Third Topic Area:**	
**Impact (benefits/drawbacks) of the e-prescribing system on personal and professional life**	
**Site C**	"How do you anticipate the e-prescribing system will impact you personally and professionally?"
	"-For example, in your interactions with patients?"
	"-Or in your interactions with colleagues?"
**Sites A and B**	"How did the e-prescribing system impact you personally and professionally?"
	"What was your learning curve like?"
**Fourth Topic Area:**	
**Facilitators and barriers to e-prescribing implementation**	
**Primarily for Float Pool Focus Group Participants**	"What do you see as the facilitators and barriers to e-prescribing implementation?"

### Data Collection

At the launch meeting, we explained the study and invited all physicians and staff to participate. Participants could then attend the focus group scheduled at their site, specific to professional role. Meetings were held at breakfast or lunchtime to facilitate participation. Three to eight participants attended each group, with the exception of the float pool group, which was attended by 22 staff. Each focus group lasted thirty minutes. At each focus group, an oral consent script was read; consent was granted by those remaining in the room (all remained). One clinician-investigator (EBD or THP) facilitated each focus group. The same research assistant (KLT) attended all focus groups, and typed anonymized discussions directly into a laptop. In total, 70 participants attended; 17 physicians and 53 staff. Between 38% (site A) and 80% (site C) of physicians participated; between 36% (site A) and 48% (site C) of staff participated. (Table 1)

### Analysis

We employed formal qualitative methods for analysis. Two coders separately coded a total of 26 pages of transcripts. One coder (EBD) was familiar with the existing literature and had led both the *a priori *mapping exercise and the development of the Focus Group Questionnaire. She was also familiar with initiatives at The Everett Clinic. When coding, she employed both deductive and phenomenologic epistemologic frameworks. [48] The other coder (ECW) was less familiar with the existing literature, and had no knowledge of The Everett Clinic. She used grounded theory techniques. [49] Each used micro-analytic techniques and axial coding to create code families, then themes. [50] Each coded to the point of theoretical saturation, that is, until no new themes emerged. Differences were resolved through discussions. We identified 142 codes and collapsed these into themes. All analyses were completed using Atlas.ti^® ^version 5.5.9 (Berlin, Germany). Our report follows the consolidated criteria for reporting qualitative research (COREQ). [51]

## Results

Ten themes emerged. Each theme describes participants' perceptions about e-prescribing implementation. We combined results from prescribers and staff within each theme, as all types of professionals were inextricably linked in the order entry process.

### Theme 1: Improved availability of clinical information resulted in prescribing efficiencies and more coordinated care

Participants noted prescribing efficiencies and more coordinated care from the improved availability of patient-specific information. Physicians anticipated forthcoming implementation of more sophisticated, patient-specific CDS alerts to guide ordering. At the same time, aware of alert fatigue, physicians expressed a desire for CDS alerts to be relevant.

"...more information about the refill request. It tells you the last visit, last lab, last refill - you don't have to take time to figure that out and it's extremely helpful." -Physician

"Drug interactions, when you add them, will be very helpful, but we do not want to be inundated with information that is mundane, such as, 'don't take with coffee'". -Physician

Staff perceived the ability to view all prescriptions as a benefit. Staff perceived this enabled them to better track controlled substances, prevent patients from requesting untimely refills, or refills from more than one prescriber concurrently.

"...to be able to get a history of prescriptions - that's incredible". - Staff

"...can track controlled substances in urgent care better." - Staff

### Theme 2: Improved documentation resulted in safer care

Participants expected the e-prescribing system to reduce medication errors and improve accuracy. At the same time, physicians perceived they needed to be cautious when prescribing, so as not to introduce computer-generated errors.

"It has turned out to do what it was billed to do - reduce/decrease errors." - Physician

"Yes, I learned that I had to be very careful if I was dictating on one patient and switched to another patient. I had to be very careful that I had the right person." - Physician

Staff perceived a diminished ability for patients to modify prescriptions, as these were sent directly to the dispensing pharmacy *via *the fax server.

"...eliminates the problem like when the doctor writes a prescription for 10 Vicodins™, and the prescription is changed to 100 Vicodins™." - Staff

### Theme 3: Efficiencies were gained by using fewer paper charts

Physicians perceived that moving from paper to e-prescribing made the prescribing process easier and quicker.

"it is much faster than waiting for the dictation to be processed." -Physician

Staff expressed doubts about ever eliminating paper charts, but perceived the benefit of fewer chart pulls and the associated time-savings.

"...there are definitely fewer chart pulls to garner information, and that saves a lot of time and money. Even receptionists can look at information and tell patients about prescriptions." -Staff

### Theme 4: Organizational support facilitated adoption

Participants perceived the importance of organizational support. Physicians appreciated the availability of technical support and requested follow-up training. They felt empowered to request improvements. One physician stated that the responsiveness of the implementation team facilitated adoption. Physicians appreciated that using the system was initially voluntary.

"The clinic as an organization provides support to you. You can also call the Help Desk or the clinical pharmacist." -Physician

"There are no mandates or ultimatums about using the computer system." -Physician

In contrast, staff wished all were required to use the system at the outset, and expressed a fear of 'hold-out' physicians.

"Not using the system is s downfall - everyone should have to use it." -Staff

### Theme 5: Transition required time; resulted in workload shift to staff

Participants acknowledged the transition would take time. They noted that entering existing prescriptions into the system was an important transition step, and perceived that efficiencies would eventually be realized.

"...learning curve - it will take time to get it going." -Physician

The burden of transition fell largely to staff, as they took responsibility for entering existing prescriptions. In essence, a workload shift occurred. Staff became facilitators of adoption, by adopting first and then assisting physicians. Physicians acknowledged the important role of staff and were appreciative of the help.

"It's a matter of getting staff to be willing to accept it and they get the providers to work with it. In a slight way, the workload is deferred from prescribers...now we're loading prescriptions into the system." -Staff

"...the medical assistants gradually got the current medications entered. If the providers had had to enter the prescriptions, it would have never happened." -Physician

### Theme 6: Hardware configurations, network stability were important in facilitating workflow

Two aspects of hardware configuration were perceived as instrumental to successful implementation - remote access from home for physicians and the availability of a personal laptop computer in each examination room. Physicians stated that remote access made access to clinical information easier, enabling them to provide better and more coordinated care. Simultaneously, they expressed concern about system security. Physicians also perceived the benefit of having the laptop computer in the examination room.

"I also like it for nighttime calls - you can see what medications the patient is on. It really helps with patient care. - Physician

"Having a record in the exam room has been a tremendous help." -Physician

Participants also perceived that they had become reliant on network stability. Staff noted the workarounds required when the system 'went down'.

"When/if the computers go down. This is when you realize that you've really adapted." -Physician

"It is more difficult when the system goes down. You have to come up with other ways to determine what the patient has been prescribed..." -Staff

### Theme 7: System use was time-neutral or time-saving

Physicians did not expect time-savings; they expected that using the system would initially take longer, especially when caring for patients with multiple, complex problems. Many were pleasantly surprised, noting that refills were quicker and easier to process. Some even noted that it shortened their day in the office.

"This has been helpful to me. I don't know that it saves me any time, but it helps." -Physician

"We were told it would help with refills - they would be easier and they have been." -Physician

"It shortens your day in clinic by allowing you to work from home." -Physician

Staff perceived the system saved time after the transition period.

*"...definitely a lot of work in the beginning, but now that we're on the one-year mark*, *wow, it's much faster!" -Staff*

### Theme 8: Changes in patient interactions enhanced patient care but required education

The effect of the e-prescribing system on patients figured prominently in discussions. Although initially, physicians feared that they would lose the eye contact usually made when handing a paper prescription to a patient, they noted this fear was not realized. They noted the e-prescribing system facilitated improved scheduling.

"...don't feel like there has been a negative interaction. I think the patients are happy." -Physician

"We like being able to see patients when they want to be seen, and also, reducing backlog." -Physician

Staff were pleased with the ease with which prescriptions could be refilled, and with which they could identify patients in need of follow-up. Staff noted the need for patient education, as they perceived that patients sometimes failed to realize their prescriptions were sent to the pharmacy ahead of them, and this created confusion.

"...expected to make it easier to do refills for patients. It has done that. We are able to catch patients who haven't been in for a long time...tell them they need to come in...." -Staff

"...patients don't understand how we're getting the prescription to their pharmacy, so patients end up waiting here for a prescription that has already been computer-faxed to their pharmacy." - Staff

### Theme 9: Pharmacy communication was enhanced but required pharmacy and patient education

Physicians were mostly silent on this theme, as communicating with pharmacies was a role that fell largely to staff. Staff initially noted that computer-faxing eliminated pharmacy-related telephone calls but later realized that educating pharmacists about the new system was necessary to smooth the prescription ordering process. Staff also noted that patients required education about the time needed for pharmacies to fill each prescription.

"I like not having to be on hold on the telephone with the pharmacist a lot." -Staff

"Some fax machines will only try to send a fax so many times...if the pharmacy line is busy, how will we know this?" -Staff

"A pharmacist says there are times...prescriptions can really stack up and when a patient gets there, it has not been filled. There needs to be patient education about giving the pharmacy time...just because it is electronic, doesn't mean it is instantaneously filled." - Staff

### Theme 10: Positive attitudes facilitated adoption

Participants' attitudes contributed to successful adoption. Many came into the project with a 'can-do' attitude. Others were more reserved, yet determined to adopt; confident that it would eventually be beneficial.

"We are expecting the change to come and that the bugs will get worked out." -Physician

"Once I got used to it, it was OK. I'm not the fastest person with new technologies, but we got the basics..." -Physician

Those who had a solid background in computer use expressed confidence in their abilities and found it easy to use.

"I used the system from the very beginning... The system I used before was different - this one is better. I've used e-prescribing since I graduated from medical school." -Physician

The strategy of implementing last at the site where the most reluctant physicians practiced seemed to help.

"...now that providers at other sites have done it, we can probably do it, too." -Physician

Overall, impressions were positive. Physicians expected the system to be useful, and had no concerns about it having a negative impact. When asked if they would recommend the system to their former medical school classmates, one physician responded,

"Yes, absolutely, I'd participate in using this system". -Physician

"We are not expecting things to be negatively impacted." - Physician

Staff expected the e-prescribing system to offer accuracy and convenience.

"Faster, more convenient, more accurate" -Staff

"It's been easier than expected." -Staff

"Do it! Absolutely! It's a good thing." -Staff

### Mapping Focus Group Results to the ITAM

**Figure 2:**
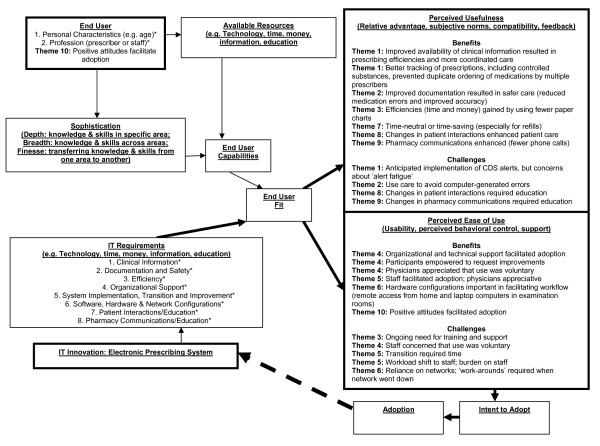
**Information Technology Adoption Model (ITAM) **[26-28]. CDS = Clinical Decision Support; IT = Information Technology; ITAM = Information Technology Adoption Model. *Indicates *a priori *information obtained from literature [31-46].

Figure 2 illustrates the mapping of our findings to the domains of the ITAM. *A priori *we populated the *End User *box with the personal characteristics and profession, and the *IT Requirements *box with a list of eight requirements necessary for successful implementation. These are indicated by the asterisks (*). Leadership anticipated and worked in advance to implement a system that met the first six of these eight requirements. The need for requirements seven and eight (education) were not as highly anticipated although they had also appeared in the literature. In conducting our analysis we mapped our themes to the ITAM, focusing on the *Perceived Usefulness *and *Perceived Ease of Use *boxes. Each theme appears at least once in the 'Benefits' and at least once in the 'Drawbacks' section of at least one of these two boxes, with the exception of Theme 7 (time-neutral or time-saving) and Theme 10 (positive attitudes facilitated adoption), which do not appear in the 'Drawbacks' section of either box. Theme 10 also maps to the *End User *box. All eight requirements in the *IT Requirements *box were carried forward to either the *Perceived Usefulness *or the *Perceived Ease of Use *box.

## Discussion

Our findings describe perceptions of physicians and staff when adopting a homegrown, EHR-based, e-prescribing system in primary care clinics in an independent, medical group. Our findings mapped logically to the ITAM where themes fit the categories of benefits and drawbacks to both *perceived usefulness *and *perceived ease of use *of the e-prescribing system, albeit with some potential overlap. As intended by leadership when specifying IT requirements, the e-prescribing system was *perceived to be useful *in creating prescribing efficiencies, more coordinated care, and safer care from improved availability and documentation of clinical information. Another initial motivator, the decreased administrative burden of paper charts, was also perceived to hold true. Another efficiency, the time-savings achieved in ordering refills, was also perceived to be useful. Not highly anticipated were the educational initiatives undertaken that were motivated by the changed way patients interacted with physicians and staff interacted with pharmacies. Both were perceived as useful and beneficial.

Contributing to *perceived ease of use *was support from organizational leadership, the availability of technical support and participant empowerment to request improvements. Interestingly, physicians thought voluntary use eased the transition, while staff thought the opposite. This reflects the burden staff felt during the transition, when entering prescriptions into the system. Importantly, staff shouldering the burden contributed to ease of use by physicians and, ultimately, to successful adoption. Two major factors contributed to ease of use and resulted in permanent changes in workflow: physicians having remote access from home and having a laptop computer (and perhaps eventually a PC) in each examination room. Both were perceived as beneficial. Finally, the positive attitude of all participants contributed to ease of use. Positive attitudes could also be thought of as personal characteristics that each user brought to bear to achieve adoption.

Challenges were also noted from both perspectives of *perceived usefulness *and *perceived ease of use*. Prescribers' awareness of the well-known issue of 'alert fatigue' predated implementation of CDS alerts. Participants were aware of computer-generated errors, but these were perceived to occur infrequently. Staff did not expect patients and pharmacies would require education to successfully interact with the changes brought on by the system. Ongoing training and support were necessary to ensure continued ease of use, as was network reliability. The two greatest drawbacks were the time required for the transition, and the burden that inputting prescriptions placed on staff. These findings reveal the importance of setting realistic expectations and timeframes for transitions to full adoption. The Everett Clinic did anticipate this; physician and staff schedules were adjusted to be less demanding during the first three months of the transition. Even so, the lesson was markedly demonstrated. Although attitudes of participants ranged from reluctant to enthusiastic, an optimistic attitude prevailed. Participants were determined to realize the benefits of the system. The strategy of implementing last at the reluctant site (Site C) paid off, as the reluctance of those at site C was overcome through the examples set by those at sites A and B. That the system held more benefits than drawbacks was clear from the majority of focus group participants. None mentioned wanting to return to the paper-based system. The focus group results proved useful in implementing the e-prescribing system in specialty clinics.

Our focus group study is the fourth in a collection of four studies we conducted during e-prescribing implementation at The Everett Clinic. Our focus group findings are largely consistent with the results of our other work. [22-25] The mention of improved medication safety, triangulates well with the findings from our first study wherein we estimated a 70% reduction (95% confidence interval (CI) 0.23, 0.40) in the adjusted odds of a medication error occurring with e-prescribing when compared to paper-based prescribing. The positive attitudes of focus group participants triangulate well with the results of our second study, wherein we administered a survey instrument - *Information Technology in Primary Care Practice *- to many of these same focus group participants. [23] The instrument was developed from the ITAM. [26-28] Domain score results revealed that intent to adopt increased significantly for both prescribers (p < 0.04) and staff (p < 0.03) at Site C between pre- and post- implementation, and perceived usefulness increased for staff (p < 0.02). The time required to e-prescribe is not heralded by participants in the focus group study. In contrast, the results from our third study revealed that the average time to e-prescribe in the examination room was 69 seconds per prescription - 25 seconds longer than to handwrite (99.5% CI 12, 38), and 24 seconds longer than to e-prescribe at offices/workstations (99.5% CI 8, 39). [23] Each calculates to 20 seconds longer per patient or approximately six minutes per prescriber, per day. There are three reasons why there are disparate results between these two studies. First, at the time of the focus group study the time increase had not yet been quantified, as the time-motion study was completed subsequent to the focus group study. Second, the benefits of increased availability of clinical information and improved safety perceived by focus group participants may have mitigated physicians' concerns about time spent e-prescribing, to the point that these were expressed as being time-neutral. Third, at Site A, focus group results reflect the use of a personal laptop carried by each physician into the examination room with him/her, rather than use of a PC computer that was eventually installed and hardwired in each examination room. What is noteworthy is that focus group results reveal that physicians were enthusiastic about using a computer in the examination room, despite the impact on workflow. We describe this impact in greater detail in our narrative of lessons learned from implementation. [25] We also narrate other aspects of successful implementation: leadership motivates implementation, an iterative roll-out, sufficient organizational and technical support, adequate communication with all stakeholders, a team-oriented culture, readily accessible training, ongoing involvement of clinicians and integrating workflow redesign into implementation. [25] Much of this narration is echoed by focus group participants.

Our findings are consistent with those of others. Barriers to full adoption are many and include immature CDS tools, limited connectivity with dispensing pharmacies, and the time-intensity of rollout. [52] The issue of alert fatigue has been well-described. [46,52-54] Computer generated errors are a constant concern. [45] The issue of the impact of CPOE, indeed of HIT initiatives in general, on workflow is increasingly being recognized as critically important. [18,55-57] The work of Niazkhani [18] and Aarts [55] suggest that CPOE implementation is complex and that it has a profound impact on collaborative workflow beyond that of the provider. Papers that explore the interaction between IT and work processes featured prominently at the Third International Conference on Information Technology in Health Care: Socio-technical Approaches, recently held in Sydney, Australia. [56]

In a large Australian teaching hospital Georgiou identified nine areas of shared concern including work practices, software/hardware, relationships/communication, education and training, inexperienced staff and de-skilling (inability to function without the system). [57] The multi-method work of the POET investigators, also conducted in the inpatient setting, identified four high-level themes that summarize perceptions of CPOE systems: 1) organizational issues, 2) clinical/professional issues surrounding customization, 3) technical/implementation issues, and 4) issues of the organization of information and knowledge. [34] They learned that a culture of collaboration and trust, and one that engages clinicians in the process is critical to successful implementation. In the ambulatory HMO setting, they identified the additional concepts of increased patient involvement, information integration across sites, having a computer in each examination room, security concerns, and the centrality of the CPOE system to work life. They noted that gradual rollout, careful project management and ongoing improvements are critical to success. [19,20] Despite the challenges, a recent survey of US physicians using commercially-based CPOE systems revealed that they believe these systems can improve safety, quality, and efficiency, and most do not wish to return to paper-based systems. [52] Because our findings are consistent with others, we posit that our findings may be of interest to other ambulatory medical groups faced with the challenges of implanting a CPOE system in the context of an EHR.

There are limitations to our work. Although all physicians and staff were invited to participate, some chose not to. The resulting discussions may have been biased in favor of or against the system. Importantly, our work is cross-sectional. Yet, our data reveal that participants at Site C who were initially reluctant, were successfully transitioning, suggesting that they would ultimately adopt the e-prescribing technology. Despite these limitations, our data are rich in the variety of implementation stages and health care professionals represented. The unique perspectives of our two coders maximized our ability to identify all relevant themes.

## Conclusions

E-prescribing/CPOE systems have potential to improve the safety and quality of patient care. Our focus group work brings the perspective of participants from an independent medical group, and suggests that e-prescribing adoption can be successful in this setting. The approach taken by our group, including communicating initial motivators, staged rollout, providing ongoing support, and empowering participants to provide feedback facilitated successful implementation. Physicians and staff worked together during the transition phase, and described the benefits of efficiency, safety and improved patient care. Important to the ambulatory setting were patient interactions, pharmacy communications, remote access, and having computers in examination rooms. Collectively, participants embraced the change, were favorably impressed with the results, and did not wish to return to the world of paper-based prescribing.

## Competing interests

The authors declare that they have no competing interests.

## Authors' contributions

EBD conceived the project idea, participated in study design, facilitated data collection, conducted the analysis, interpreted the results, and drafted the manuscript. ECW conducted the analysis, interpreted the results, and revised the manuscript for critically important intellectual content. DPM, DFS, and PTH interpreted the data and revised the manuscript for critically important intellectual content. THP participated in data collection and revised the manuscript for critically important content. SDS was responsible for study design and revised the manuscript for critically important intellectual content. All authors have read and approved the final version of the manuscript.

## Pre-publication history

The pre-publication history for this paper can be accessed here:

http://www.biomedcentral.com/1472-6947/10/72/prepub
